# Bayes and Empirical Bayes Estimators of Abundance and Density from Spatial Capture-Recapture Data

**DOI:** 10.1371/journal.pone.0084017

**Published:** 2013-12-27

**Authors:** Robert M. Dorazio

**Affiliations:** U.S. Geological Survey, Southeast Ecological Science Center, Gainesville, Florida, United States of America; Pennsylvania State University, United States of America

## Abstract

In capture-recapture and mark-resight surveys, movements of individuals both within and between sampling periods can alter the susceptibility of individuals to detection over the region of sampling. In these circumstances spatially explicit capture-recapture (SECR) models, which incorporate the observed locations of individuals, allow population density and abundance to be estimated while accounting for differences in detectability of individuals. In this paper I propose two Bayesian SECR models, one for the analysis of recaptures observed in trapping arrays and another for the analysis of recaptures observed in area searches. In formulating these models I used distinct submodels to specify the distribution of individual home-range centers and the observable recaptures associated with these individuals. This separation of ecological and observational processes allowed me to derive a formal connection between Bayes and empirical Bayes estimators of population abundance that has not been established previously. I showed that this connection applies to *every* Poisson point-process model of SECR data and provides theoretical support for a previously proposed estimator of abundance based on recaptures in trapping arrays. To illustrate results of both classical and Bayesian methods of analysis, I compared Bayes and empirical Bayes esimates of abundance and density using recaptures from simulated and real populations of animals. Real populations included two iconic datasets: recaptures of tigers detected in camera-trap surveys and recaptures of lizards detected in area-search surveys. In the datasets I analyzed, classical and Bayesian methods provided similar – and often identical – inferences, which is not surprising given the sample sizes and the noninformative priors used in the analyses.

## Introduction

In capture-recapture and mark-resight surveys, movements of individuals both within and between sampling periods can alter the susceptibility of individuals to detection over the region of sampling. In these circumstances spatially explicit capture-recapture (SECR) models, which incorporate the observed locations of individuals, allow population density and abundance to be estimated while accounting for differences in detectability of individuals.

A variety of SECR models have been developed to accommodate different kinds of sampling protocols and capture methods [1]. The spatial point process used to model the distribution of individual home-range centers is an important component of these models. This process determines the expected size of the population within any finite region, and it specifies how the expected density of individuals is assumed to vary across the region. Many SECR models use a Poisson point process to specify the spatial distribution of home-range centers [2]–[7]. The parameters of these models have been estimated using classical statistical methods (maximum likelihood), and freely available software exists to fit these models (programs DENSITY [8] and secr (written using the R software program [9]). Other SECR models use a binomial point process to specify the spatial distribution of home-range centers [10]–[16]. In these models the number 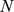 of home-range centers located within an arbitrarily large (but finite) region is assumed to be constant, unlike the Poisson point-process models wherein 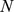 is a random outcome. By assuming a constant value for 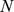, the binomial point-process models can be fitted using Bayesian methods and data augmentation [17]. In this approach the actual data set is augmented with a known number of ''all zero'' detection histories and a zero-inflated version of the base SECR model is fitted to the augmented data set. These models have been implemented using freely available software (programs WinBUGS (http://www.mrc-bsu.cam.ac.uk/bugs/winbugs/contents.shtml) and JAGS (http://mcmc-jags.sourceforge.net)). Program SPACECAP provides a more specialized implementation of this approach [18].

If the region occupied by the population is sufficiently large, the Poisson limit theorem suggests an asymptotic equivalence between the Poisson and binomial SECR models and their estimators of 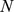. For example, in a Bayesian analysis using data augmentation, a 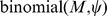 distribution is used to specify prior uncertainty in 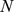. In this context the 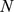 individuals may be viewed as a subset of 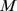 individuals whose home-range centers are distributed in a region 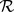 that encompasses the region 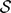 occupied by the subpopulation of size 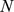. Suppose a homogeneous binomial point process is used to model the home-range centers of all 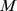 individuals; then 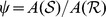, where 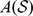 and 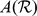 denote the respective areas of the two regions. The Poisson limit theorem establishes that if the both 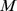 and 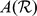 tend to infinity while keeping the binomial mean 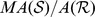 constant, the asymptotic distribution of 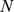 is Poisson with mean 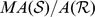. The intensity of this point process is 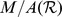, so the expected value of 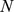 equals the product of the intensity and 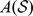, as in a homogeneous Poisson process.

The asymptotic equivalence of binomial and Poisson SECR models may not apply in small regions or small populations. In a simulation study where recapture locations were simulated for area-search surveys [7], maximum-likelihood estimates (MLEs) of population density obtained by fitting a Poisson point-process model sometimes exhibited lower relative bias than Bayesian estimates obtained by fitting a binomial point-process model. The differences in bias were most pronounced at the lowest (true) population densities, regardless of whether the posterior mean or mode was used as a Bayesian estimator of density. Similarly, the frequentist coverage of Bayesian credible intervals for population density was sometimes less than the nominal level, whereas the coverage of classical confidence intervals approximated the nominal level in the same simulation scenarios.

The source(s) of the apparent difference in performance of classical and Bayesian estimators of density cannot be inferred from this simulation study. Were the differences associated with differences in modeling assumptions (binomial vs. Poisson) or with differences in methods of inference and point estimators (MLE vs. posterior mean or mode)? Also, owing to the sizeable difference in number of data sets analyzed in each simulation scenario (the binomial model was fitted to 100 data sets whereas the Poisson model was fitted to 1000 data sets), the estimated bias and coverage of the Bayesian estimators may have included substantial Monte Carlo error, casting doubt on the statistical significance of the reported difference in performance of classical and Bayesian methods.

To shed light on this issue and to establish a formal relationship between classical and Bayesian estimators of abundance, I developed two Bayesian models of SECR data using Poisson point processes. One model is for the analysis of recaptures observed in trapping arrays; the other is for the analysis of recapture locations observed in area searches. In formulating these models I used distinct submodels to specify the distribution of individual home-range centers and the observable recaptures associated with these individuals. This separation of ecological and observational processes allowed me to derive a formal connection between Bayes and empirical Bayes estimators of population abundance that has not been established previously. I showed that this connection applies to *every* Poisson point-process model of SECR data and provides theoretical support for an estimator of abundance proposed for recaptures in trapping arrays [19].

To illustrate the results of both classical and Bayesian methods of analysis, I compared Bayes and empirical Bayes esimates of abundance and density for both simulated and real populations of animals. Surprisingly few analyses of SECR data have assumed spatial variation in individual density [19]–[21]; therefore, I analyzed recaptures of animals in a simulated trapping array to illustrate how maps of individual density may be estimated for a landscape containing substantial habitat heterogeneity. I also repeated the simulation study of recaptures in area-search surveys [7]. In contrast to [7], I analyzed a larger number of data sets using only Poisson point-process models (classical and Bayesian) and I evaluated the amount of Monte Carlo error in the estimates of bias and frequentist coverage. To illustrate analyses of real data with the Poisson point-process models, I used two iconic datasets: recaptures of tigers detected in camera-trap surveys [12], [13], [17] and recaptures of lizards detected in area-search surveys [7], [10]. I conclude the paper with a discussion of the relative merits of classical and Bayesian SECR models and a description of some useful extensions of these models.

## Methods of Analysis for Spatial Capture-Recapture Data

In this section I describe two SECR models, one for the analysis of recaptures observed in trapping arrays and another for the analysis of recaptures observed in area searches. These models can be specified hierarchically using one component to describe the process that generates the spatial distribution of individuals in the population and a second component to describe the process that leads to an observable sample of individuals. The first component, a Poisson point-process model, is identical for both SECR models; therefore, I describe this component first and follow with descriptions of the underlying assumptions and likelihood functions of the two observation models.

### Poisson Point-process Model of Individual Activity Centers

This component of the SECR models is similar to the model of home-range centers (more generally, activity centers) proposed by [2] and extended by [3]. Consider a population of individuals that reside within a bounded, geographic region 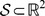 that has scientific or operational relevance. Each individual is assumed to move randomly around its activity center 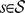, so that the spatial distribution of activity centers defines the population.

I assume that the activity centers are a realization of an inhomogeneous Poisson point process parameterized by a first-order intensity function 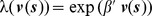 that includes a vector of regressors 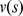 at location 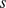 and a vector of parameters 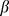. (I use a prime symbol to denote the transpose of a matrix or vector.) To simplify notation, I will use 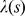 instead of 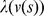 to express the implied functional dependence of 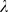 on location 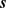.

In the context of SECR models, 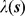 denotes the limiting, *expected* density of individuals (number of individuals per unit area) at location 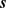. That is, for a small region 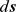 of area 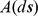 centered at 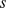,
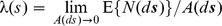
for the Poisson point process 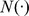 [22]. Furthermore, the number 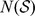 of individuals in 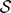 – that is, the size of the population – is a Poisson random variable that depends on the mean intensity of the process over region 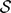: 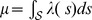. In other words, 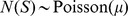. Given a realized population of size 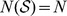, the 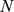 activity centers are independent and distributed with probability density function 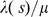. Therefore, under the assumptions of the point-process model the conditional density of the 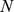 locations is



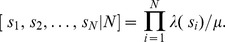



(Note that I specify probability density functions using bracket notation wherein 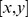 denotes the joint density of random variables 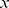 and 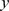, 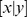 denotes the conditional density 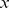 given 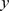, and 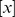 denotes the unconditional (marginal) density of 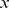 [23]. In addition, I do not distinguish random variables and their realized values by uppercase and lowercase notations. This practice, while common in Statistics, can be cumbersome and can produce notations that are unconventional in Ecology.)

An important, special case of the model of activity centers is the *homogeneous* Poisson point process, wherein 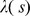 is assumed to be constant at all locations (i.e., 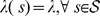). For this model, the expected size of the population depends only on 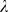 and on the area 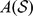 of region 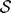 because 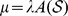. In addition, the probability density of each activity center is constant since 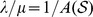. In other words, under the assumptions of the homogeneous model, the activity centers are independently and uniformly distributed over region 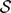.

### Analysis of Recaptures at Trapping Array Locations

In this section I describe a model for recaptures of individuals that may be detected at each of 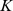 distinct trapping locations. These locations are often selected to form an array (or grid) of uniformly spaced traps. In practice, these traps often correspond to recording devices (such as cameras or people capable of detecting the presence of uniquely marked individuals) as opposed to devices that capture and retain individuals. [5] have proposed the term ''proximity detectors'' for these kinds of traps. Throughout this paper, I use the words, *detections* and *captures*, interchangeably to describe observations of marked individuals.

I assume an individual may be detected at the *k*th trap during each of 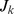 trapping periods of equal duration. Although multiple detections of an individual at the same trap are possible and easily modeled [12], I consider here a model in which individuals are assumed to be detected only once per trapping period at any trap. Specifically, I assume that each detection of an individual at trap 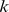 is an independent Bernoulli outcome in which capture probability 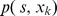 is a function of the Euclidean distance between an individual's activity center 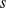 and the location 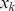 of trap 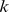. In addition, I assume that recaptures of an individual in different traps occur independently.

Several functional forms for 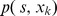 are possible [5]; here, I consider a function based on the Gaussian kernel:
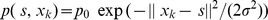
where 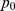 denotes the maximum probability of capture (applicable when 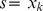) and where 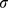 denotes a positive-valued scale parameter. If movement of individuals is the mechanism that makes them susceptible to detection, the scale parameter essentially quantifies the spatial extent of movements around the activity centers; however, this distance-based detection function is also applicable in surveys where individual movements are not responsible for differences in capture probability [1]. The essential feature is that 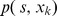 is highest when locations 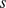 and 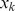 coincide and declines monotonically as the distance between 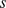 and 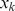 increases.

To complete the model description, let 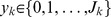 denote the number of trapping periods in which an individual is detected at the *k*th trap. A sum of 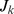 independent and identically distributed Bernoulli random variables has a binomial distribution; therefore, 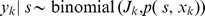. Moreover, since recaptures of the same individual in different traps are assumed to occur independently, the joint probability of these observations (conditional on the individual's activity center 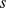) equals the product of trap-specific, binomial probabilities:
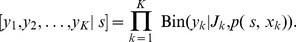



(Probability density functions for the binomial, Bernoulli, and normal distributions are abbreviated using Bin, Bern, and N, respectively (as in tables 2.1 and 2.2 of [24]).)

We are now equipped with all of the components needed to derive the likelihood function of the model. Let 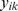 denote the number of detections of the 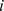
th individual at trap 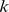 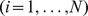. For the moment, suppose (counterfactually) that each of the 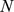 individuals is detected at least once during trapping. In this case the likelihood function for the unknown parameters 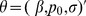 equals the joint density of 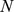 and the 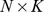 matrix 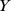 of individual- and trap-specific counts:
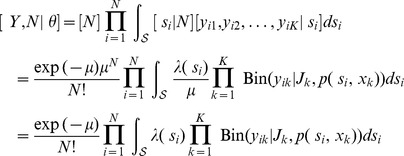



In classical (non-Bayesian) statistics, the likelihood function is often written as a function of 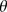 (for example, 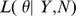), emphasizing that 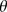 is the unknown parameter to be estimated given data. I depart from this convention here for reasons that will become clear shortly; however, it should be understood that 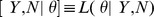. The contribution of the 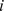
th individual to this likelihood function is based on the marginal probability of the observed counts, 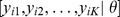, obtained by integrating the individual's activity center 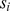 (an unobserved random variable) from the joint density of 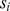 and the observed counts. Although the integrals for 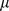 and each individual's contribution to 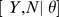 cannot be expressed in closed form, these integrals can be evaluated numerically by partitioning region 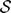 into a sufficiently fine grid and evaluating a Riemann sum.

In reality, only 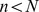 individuals are detected during trapping, and our objective is to estimate 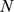 and the parameters 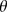 given the number of observed individuals (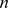) and their 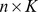 matrix 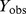 of trap-specific counts. Let 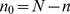 denote the unknown number of individuals that are *not* detected in any of the 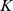 traps. For these individuals, 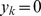 (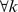). The previous likelihood function must be modified to account for the unobserved individuals in the population. Given our modeling assumptions, the probability that an individual in the population was unobserved is
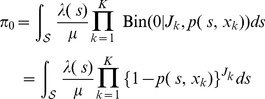
(1)


The likelihood function of the ''complete data'' (wherein 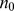 is treated as observable) is
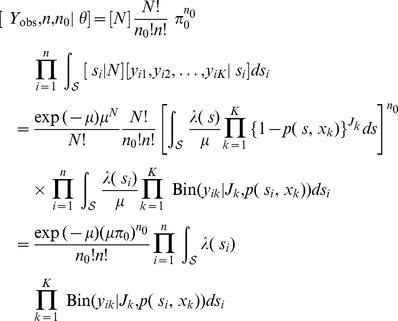
which accounts for the 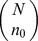 ways that 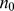 distinct individuals may remain unobserved after sampling a population of size 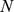. We marginalize 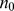 (by summation) from this ''complete-data'' likelihood to obtain the likelihood function of the observable data:



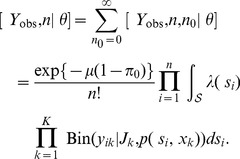
(2)The maximum-likelihood estimator (MLE) of 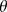 cannot be expressed in closed form; however, numerical maximization of (2) is straightforward and allows a maximum-likelihood estimate 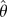 to be calculated for any data set. Asymptotic variances and covariances of the model's parameters may be estimated by inverting the observed information matrix.

#### Empirical bayes estimator of abundance

To develop an estimator of population size 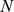 using classical (non-Bayesian) methods of analysis, we use a single application of Bayes' rule to derive the conditional probability of 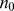 given the observed data and the parameters 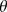:
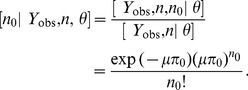
(3)


Therefore, the conditional distribution of 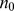 is 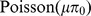 and depends on the observed data (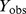 and 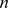) only indirectly in the sense that 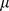 and 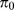 are functions of 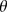, which must be estimated. The mean of the conditional distribution of 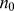 provides the basis for the following empirical Bayes estimator of 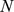:
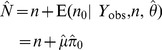
(4)where 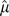 and 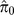 correspond to the values of 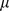 and 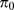 evaluated at the estimate 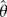.

To estimate 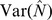, we must account for the expected variance of 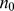 based on its conditional (Poisson) distribution and for the uncertainty involved in estimating 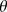. I derived the following estimator of 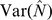 by adopting the conceptual framework of [25] and by using the delta method to propagate the uncertainty of 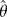:
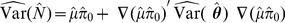
(5)where 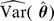 denotes the estimated asymptotic variance-covariance matrix of 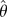 and where 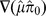 denotes the gradient of 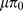 with respect to 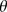 evaluated at 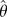. (See Appendix S1 for the derivation of (5).) A 95% confidence interval for 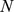 may be computed by assuming that the sampling distribution of the estimates of 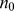 is lognormal with mean 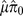 and variance 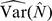.

Though not entirely obvious, it's worth noting that empirical Bayes estimates of 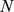 and their variances increase automatically with the size of region 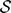. This occurs because the integral in (1) increases with the size of 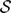. In addition, the abundance of activity centers located within any subset of 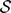 may be estimated using 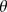 without additional computation owing to the independence of events of the Poisson process over 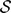 [22].

#### Bayes estimator of abundance

To develop an estimator of population size 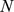 using Bayesian methods of analysis, we must extend the model to specify uncertainty in the magnitude of 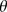 *prior* to observing the data. To do this we combine the complete-data likelihood function with a prior density 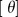 to form the (unnormalized) posterior density:
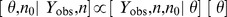



Note that the parameters of this model include both 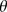 and 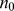. We could instead have chosen the likelihood of the observed data (Equation 2) and obtained the marginal density 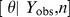 of this posterior directly; however, retaining 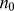 as a parameter actually simplifies the Markov chain Monte Carlo (MCMC) algorithm used to fit the model (see Appendix S1).

Since 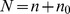, Bayesian inferences about 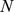 are based entirely on the posterior distribution of 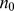, which has probability mass function
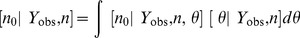
(6)


Note that the integrand in this equation contains the conditional posterior probability of 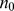, which was derived earlier (Equation 3) and used to compute the empirical Bayes estimator of 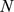. In contrast to that estimator, Bayesian inferences about 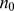 – and therefore, about 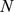 – integrate over the posterior uncertainty in 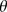 so that additional calculations (as in (5)) are not needed to estimate the uncertainty in 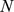 correctly. Summaries of the posterior distribution of 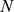 (e.g., mean, mode, quantiles, etc.) may be estimated directly from (6). In addition, these estimates are valid for any sample size and do not rely on asymptotic approximations, unlike the empirical Bayes confidence intervals. Of course, the price of that validity is having to specify a prior distribution for 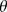, which may influence estimates of 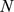 if relatively few individuals have been recaptured.

### Analysis of Recapture Locations Observed in Area Searches

In this section I describe a model for recaptures of individuals that may be detected during each of 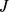 uniform searches of a sampling region 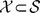. During these area-search surveys, as they have come to be called, individuals may be detected but their capture locations are not fixed – unlike the recaptures at trapping arrays. In fact, in an area search the capture location of an individual is not even observable if the individual is not detected. Therefore, the detections of individuals and their capture locations are both stochastic outcomes of area-search surveys.

To specify a model of the observable capture (and recapture) locations, I assume that each individual moves randomly around its activity center 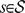. Specifically, I use a bivariate normal distribution to model the locations that arise from each individual's movements. Let 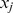 denote a random variable for the location of an individual during the 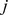
th area-search survey (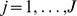). Under the bivariate normal model, 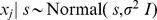, where 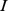 denotes a 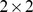 identity matrix. Note that whereas all individuals share the same scale parameter 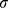, which quantifies the extent of their movements, the locations at which a single individual might be observed depend on its activity center 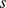. [10] and [7] also used the bivariate normal distribution to specify models of area-search data; however, alternative movement models are also possible. The essential feature of the SECR model developed here is the conditional dependence between an individual's locations and its activity center.

To complete the model, we need to specify the process that leads to detections of individuals. Let a random variable 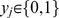 denote whether an individual is detected (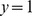) or not (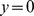) during the *j*th search of region 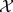. The value of 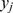 clearly depends on an individual's location 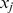 during the *j*th survey (since 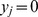 if 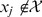); therefore, I assume
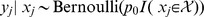
where 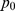 is the probability that an individual present in region 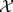 is detected during a single search of the region. (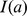 denotes the indicator function, which equals 1 if argument 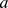 is true and 0 otherwise.).

The locations and detections of the same individual in different surveys are assumed to occur independently; therefore, the joint density of these observations (conditional on the individual's activity center 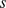), equals the product of survey-specific densities:
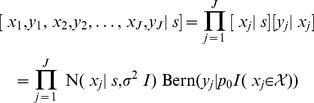
(7)


Recall, however, that an individual's capture location is not observable in surveys where the individual is not detected; therefore, the joint density in (7) cannot be used in the likelihood function of the observable data. Instead, we must factor the joint density into two components, one for the locations of individuals that are detected and another for the marginal probability of the non-detections obtained by integrating the unobserved recapture locations from (7).

The first component of the joint density is associated with the value of 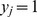 (detection). This observation implies 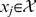; thus, the joint density of 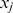 and 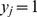 is
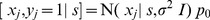
(8)


The second component of the joint density is associated with the value of 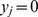 (non-detection). In this case the individual's location is unobserved, so the marginal probability of non-detection is
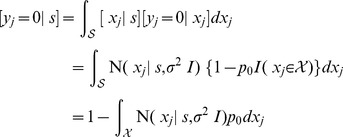
(9)


Note that (9) is based on the assumption that 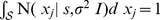, which implies that an individual's locations are contained within region 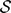 during the area-search surveys of 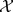. Technically, and depending on the value of 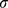, this assumption could be violated for individuals whose activity centers are located near the boundary of 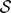. However, the consequences of these “edge effects” for estimation usually can be minimized simply by increasing the size of 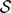, which reduces the relative contribution of these individuals to the likelihood function. If this corrective measure does not solve the problem, then 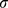 may be so high relative to the size of 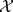 that movements of individuals reduce the chances of multiple detections (recaptures of the same individual) and the problem is not tractable anyway [7], [26].

We now have all of the elements needed to derive the likelihood function of the model. Let 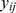 indicate whether the 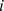
th individual is detected or not during the *j*th search of region 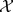 (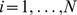). Similarly, let 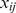 denote the location of the *i*th individual during the *j*th area-search survey. Our objective is to estimate 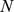 and the parameters 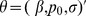 given the number of observed individuals (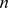), the 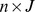 matrix 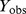 of detection indicators, and the partially observed 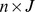 matrix 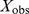 of locations. Let 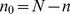 denote the unknown number of individuals that are *not* detected during the 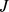 area-search surveys. For these individuals, 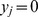 (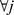). As before, the likelihood function of the observable data must account for the unobserved individuals in the population. Given our modeling assumptions, the probability that an individual in the population was unobserved is
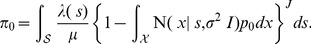
(10)


The likelihood function of the “complete data” (wherein 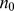 is treated as observable) is
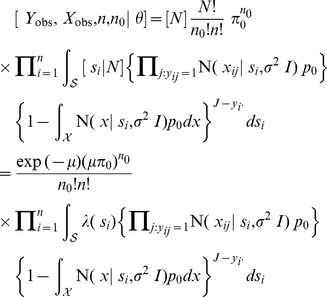
where 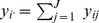 is the number of area-searches in which the 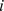
th individual is detected. We marginalize 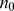 (by summation) from this “complete-data” likelihood to obtain the likelihood function of the observable data:



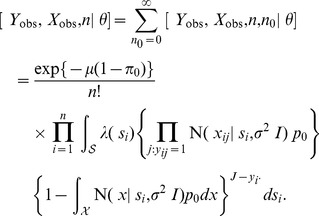



The maximum-likelihood estimator (MLE) of 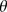 cannot be expressed in closed form; however, numerical maximization of the likelihood function is straightforward. Although the numerical integrations over 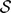 require discretization (as described earlier) and can be computationally intensive, efficient algorithms exist for integrating the bivariate normal density function over 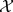. Asymptotic variances and covariances of the model's parameters may be estimated by inverting the observed information matrix.

#### Estimators of abundance

Classical (non-Bayesian) and Bayesian estimators of population size 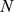 may be derived using the same approach taken with the trapping array model. For example, a single application of Bayes' rule reveals that the conditional distribution of the number of unobserved individuals in the population is Poisson:
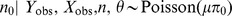
where 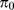 is defined in (10). The mean of this distribution provides the basis for the empirical Bayes estimator of 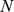: 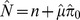, where 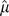 and 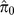 correspond to the values of 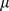 and 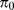 evaluated at the maximum likelihood estimate 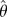. Similarly 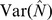 is estimated using the empirical Bayes estimator (5).

To develop the Bayesian estimator of 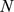, we combine the complete-data likelihood function with a prior density 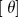 to obtain the (unnormalized) posterior density:
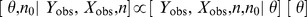



The MCMC algorithm used to fit this model is given in Appendix S1. Since 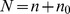, Bayesian inferences about 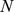 are based entirely on the posterior distribution of 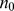, which has probability mass function
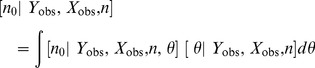
(11)


Summaries of the posterior distribution of 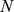 may be estimated directly from (11), as described earlier.

## Analyses of Real and Simulated Data

### Trapping-array Surveys in a Spatially Heterogeneous Habitat

**Figure 1:**
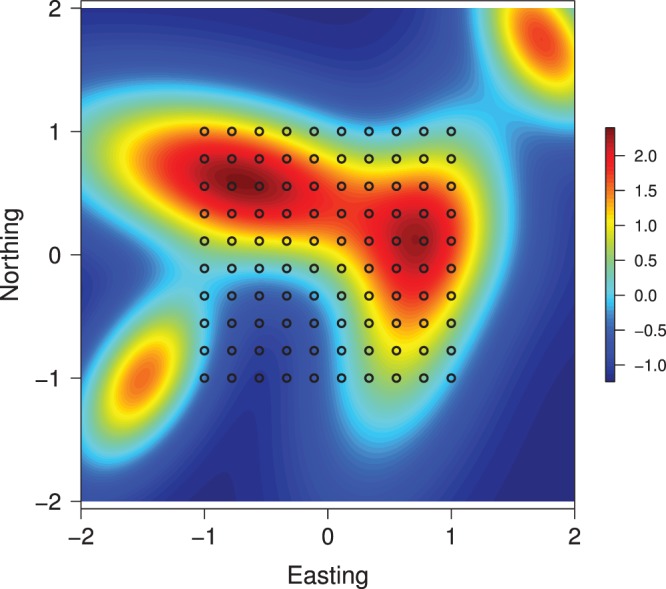
Map of spatial variation in habitat quality for a simulated population of individuals exposed to an array of 100 traps. Trap locations are superimposed on map.

The density of individuals can vary substantially in regions of spatially varying habitat. To mimic this situation, I simulated a population of individuals living in a square (2 km 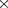 2 km) region with relatively large differences in habitat quality (Figure 1). The limiting expected density of individuals in this region was assumed to vary as a loglinear function of habitat quality 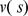 as follows: 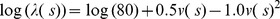, where 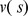 was centered and scaled to have mean zero and unit variance. Individuals were detected in an array of 100 traps placed within a 1 km 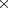 1 km square located at the center of the region (Figure 1). I assumed that individuals possessed unique marks for identification and may have been detected during each of five survey periods, as in camera-trap surveys. Maximum detection probability 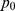 was assumed to be 0.25, and the scale parameter 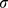 was assumed to be 0.4 km.

Of the total population of 643 individuals, 535 were observed in the trapping-array surveys. Many of these individuals were detected at more than one trap (average = 7.8 traps per individual), but few individuals were observed more than twice at the same trap (average = 1.2 detections per trap).

I fitted the SECR model for recaptures in trapping arrays to these data by partitioning the region occupied by the population into a grid of square pixels (width = 160 m) for numerical integration. Further reductions in pixel width did not change the value of the maximized log likelihood. I used maximum-likelihood estimates of the model's parameters to initialize the Markov chain used in the Bayesian analysis. In addition, I used 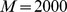 successive draws of the Gibbs sampler (Appendix S1) to estimate posterior means and quantiles of the model parameters. Monte Carlo standard errors of posterior means and quantiles were computed using the subsampling bootstrap method [27], [28] with overlapping batch means of size 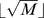.

**Figure 2:**
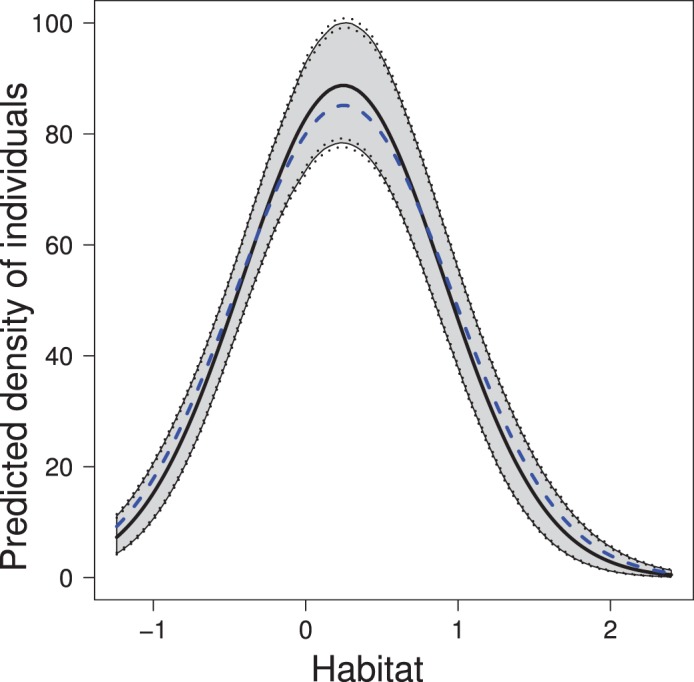
Predicted mean density of individuals in a simulated population as a function of habitat quality. Shaded region indicates 95% credible interval. Dotted lines indicate uncertainty of upper and lower credible limits due to Monte Carlo error. Blue dashed line indicates the true relationship between 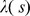 and habitat quality.

**Figure 3:**
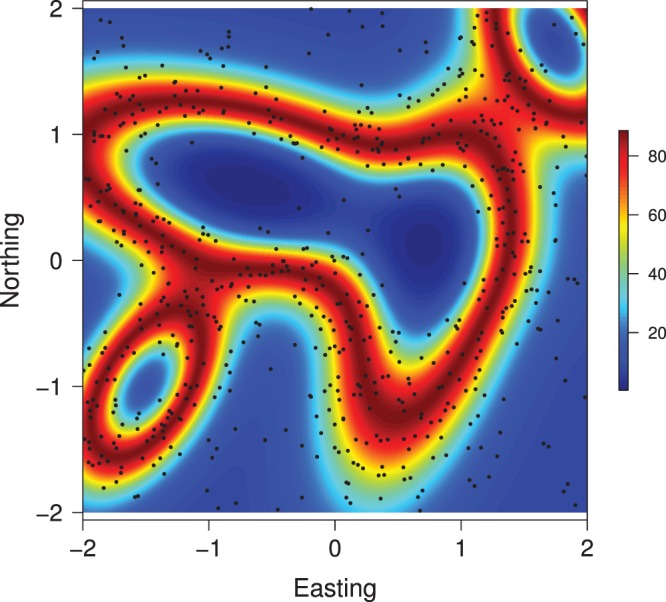
Map of predicted mean density of individuals in a simulated population. Activity centers of individuals are superimposed on the map.

**Figure 4:**
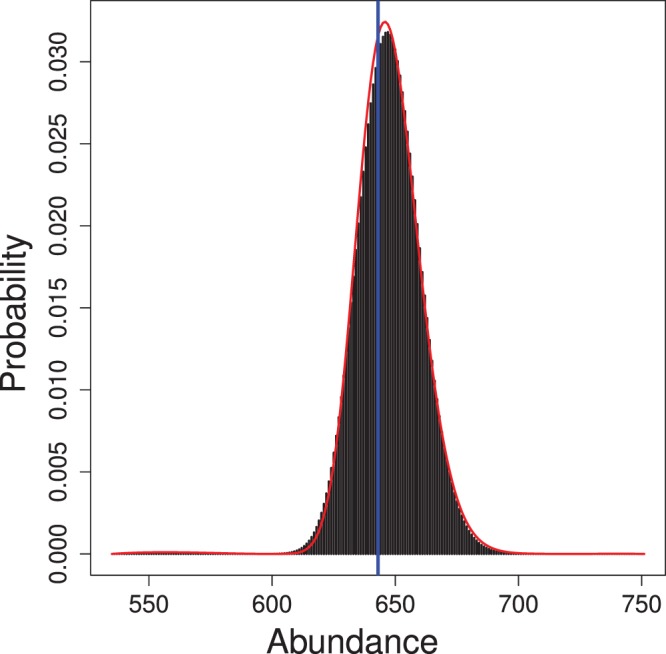
Posterior distribution of abundance of simulated population. Red line indicates the estimated sampling distribution of empirical Bayes estimates of abundance. Vertical blue line indicates true population size.

Classical and Bayesian estimates of the model parameters and of their 95% confidence or credible intervals are quite similar (Table 1). Strong agreement also exists between the parameter estimates and the parameter values used to generate the data, which is not surprising given the size of the population and sample. This agreement is manifest in predictions of 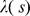 as a function of habitat quality (Figures 2 and 3). The empirical Bayes and Bayes estimates of population size 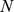 are essentially identical (647.8 and 647.6 individuals, respectively) given the Monte Carlo error of the Bayes estimate. The 95% confidence and credible intervals for 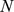 are also quite similar since the posterior distribution appears to be approximately lognormal (Figure 4).

**Table 1:** Estimates of parameters of model fitted to recaptures of individuals in a simulated population.

	Maximum-likelihood estimates			Bayesian estimates		
Parameter (units)	Estimate	2.5%	97.5%	Mean	2.5%	97.5%
*β*_0_	4.412	4.292	4.532	4.415 (0.0030)	4.297 (0.0047)	4.531 (0.0044)
*β*_1_	0.557	0.359	0.755	0.563 (0.0044)	0.370 (0.0061)	0.784 (0.0061)
*β*_2_	−1.123	−1.353	−0.892	−1.143 (0.0067)	−1.367 (0.0091)	−0.911 (0.0091)
*p*_0_	0.254	0.244	0.263	0.254 (0.0002)	0.245 (0.0003)	0.263 (0.0003)
σ (km)	0.400	0.393	0.407	0.400 (0.0002)	0.393 (0.0003)	0.407 (0.0003)
*N* (individuals)	647.8	625.3	674.2	647.6 (0.26)	624 (0.45)	672 (0.62)

*N* is the abundance of individuals in the population. Lower and upper limits of 95% confidence intervals (based on ±1.96 asymptotic standard errors) and of 95% credible intervals are given in the columns labeled 2.5% and 97.5%, respectively. Monte Carlo standard errors are given in parentheses.

### Camera-trap Surveys of Tigers

I fitted the SECR model for recaptures in trapping arrays to a set of data that have been analyzed previously using both non-spatial and spatial capture-recapture models [12], [13], [17]. [12] provide a detailed description of these data. Briefly, the data include photographic recaptures of 44 tigers that were exposed to a spatial array of 120 camera traps established within the Nagarahole Reserve in the state of Karnataka, southwestern India [12]. The identity of each tiger could be established from photographs because these animals possess natural marks (unique stripe patterns). All 120 camera traps were not operated simultaneously. Instead, the reserve was partitioned into four subregions, each containing about 30 traps, and tigers were photographed in each subregion during each of 12 consecutive days. Because individual tigers were rarely detected more than once in the same day and trap, I treated multiple detections on the same day and trap as a single recapture, in accordance with the underlying assumptions of the SECR model. [12] made the same assumption, treating the observations of each tiger as a sequence of binary encounters over 12 days of trapping.

In the absence of spatial covariates of tiger density, I fitted the model based on a homogeneous Poisson point process using both classical and Bayesian methods of analysis. The minimum rectangular region needed to encompass the entire trapping array spanned an area of 772 km^2^ (20.7 km × 37.3 km). To fit the model, I assigned 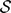 to be the rectangular region that buffers this minimum rectangle by 12.5 km on each side, and I partitioned 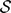 into a grid of square pixels (width = 0.5 km) for numerical integration. Further increases in the size of 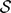 and further reductions in pixel width did not change the value of the maximized log likelihood. To conduct a Bayesian analysis of the data, I used maximum-likelihood estimates of the model's parameters to initialize the Markov chain and I used 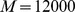 successive draws of the Gibbs sampler (Appendix S1) to estimate posterior means and quantiles of the model parameters. Monte Carlo standard errors of posterior means and quantiles were computed as described earlier.

**Figure 5:**
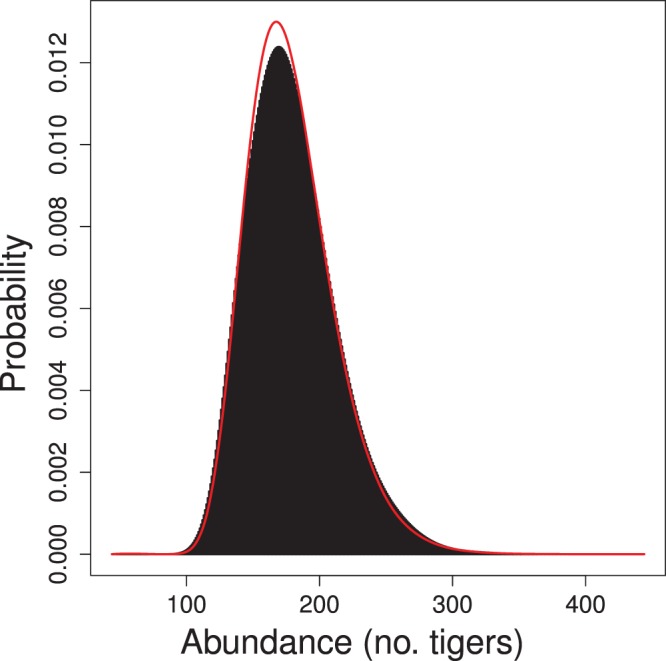
Posterior distribution of abundance of tigers in a rectangular region of area 1452^2^. Region is defined as trapping rectangle buffered by 5

Classical and Bayesian estimates of the model parameters and their 95% confidence or credible intervals are quite similar (Table 2). The average density of tigers is estimated to be 0.123 individuals km^−2^. The probabilities of detecting these tigers appears to be very low since the estimated maximum detection probability is only about 0.02. For comparison with a previous analysis of these data [17], I estimated the number of tigers present in a rectangular region that buffers the minimum rectangle by 5 km on each side (area = 1452 km^2^). [17] incorrectly reported this region as buffering the minimum rectangle by 7.5 km on each side (area = 1866 km^2^) (Royle, personal communication). The empirical Bayes and Bayes estimates of 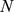 in this region are essentially identical (178.8 and 179.2 tigers, respectively) given the Monte Carlo error of the Bayes estimate. The 95% confidence and credible intervals for *N* are also quite similar since the posterior distribution appears to be approximately lognormal (Figure 5).

**Table 2:** Estimates of parameters of model fitted to recaptures of tigers at trap locations.

	Maximum-likelihood estimates			Bayesian estimates		
Parameter (units)	Estimate	2.5%	97.5%	Mean	2.5%	97.5%
*λ* (tigers km^−2^)	0.123	0.083	0.182	0.123 (0.0013)	0.082 (0.0011)	0.180 (0.0020)
*p*_0_	0.016	0.009	0.027	0.017 (0.0002)	0.009 (0.0002)	0.027 (0.0003)
σ (km)	1.95	1.57	2.44	1.95 (0.0087)	1.59 (0.0080)	2.46 (0.0168)
*N* (tigers)	178.8	125.6	254.1	179.2 (1.64)	124 (1.20)	256 (2.68)

*N* is the abundance of tigers in a rectangular region of area 1452 km^2^ (trapping rectangle buffered by 5 km). Lower and upper limits of 95% confidence intervals (based on ±1.96 asymptotic standard errors) and of 95% credible intervals are given in the columns labeled 2.5% and 97.5%, respectively. Monte Carlo standard errors are given in parentheses.

### Area-search Surveys of Lizards

I fitted the SECR model for area-search surveys to a set of data that have been described and analyzed previously [7], [10]. The data include the recaptures of 68 flat-tailed horned lizards (*Phrynosoma mcallii*) within a 9-ha square plot (width = 300 m) located in southwestern Arizona, USA. During each survey, this plot was searched thoroughly for lizards, which were marked and released after noting their capture locations. A total of 14 surveys were conducted over 17 days producing 134 captures [7].

In the absence of spatial covariates of lizard density, I fitted the model based on a homogeneous Poisson point process using both classical and Bayesian methods of analysis. To fit the model, I assigned 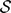 to be the square region that buffers the surveyed plot by 150 m on each side, and I partitioned 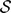 into a grid of square pixels (width = 5 m) for numerical integration. Further increases in the size of 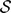 and further reductions in pixel width did not change the value of the maximized log likelihood. To conduct a Bayesian analysis of the data, I used maximum-likelihood estimates of the model's parameters to initialize the Markov chain and I used 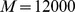 successive draws of the Gibbs sampler (Appendix S1) to estimate posterior means and quantiles of the model parameters. Monte Carlo standard errors of posterior means and quantiles were computed as described earlier.

**Figure 6:**
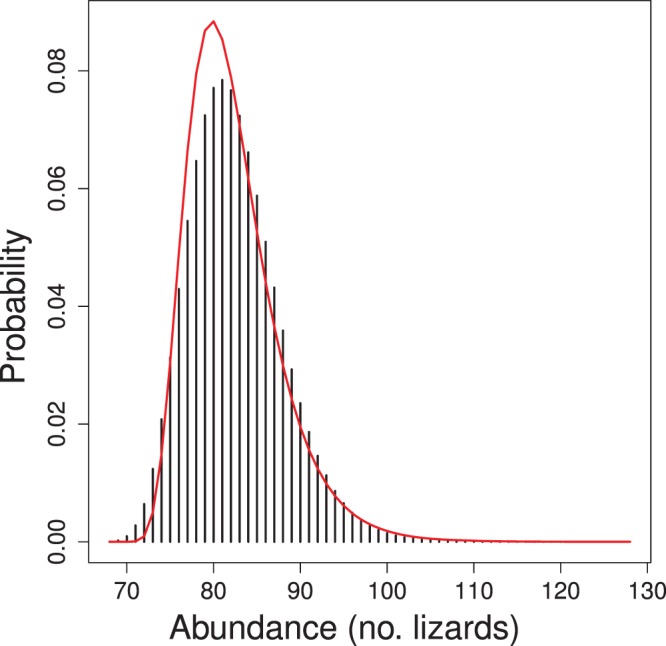
Posterior distribution of abundance of lizards in the 9 ha search area. Red line indicates the estimated sampling distribution of empirical Bayes estimates of abundance.

Classical and Bayesian estimates of the model parameters and their 95% confidence or credible intervals are quite similar (Table 3). The average density of lizards is estimated to be 8.06 individuals ha^−1^. For comparison with previous analyses of these data, I estimated the number of lizards present in the 9-ha surveyed plot. The empirical Bayes and Bayes estimates of 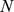 in the plot are essentially identical (82.3 and 82.5 lizards, respectively) given the Monte Carlo error of the Bayes estimate. The 95% confidence and credible intervals for 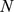 are also similar (Figure 6).

**Table 3:** Estimates of parameters of model fitted to locations of lizards recaptured during area-search surveys.

	Maximum-likelihood estimates			Bayesian estimates		
Parameter (units)	Estimate	2.5%	97.5%	Mean	2.5%	97.5%
*λ* (lizards ha^−1^)	8.06	6.23	10.43	8.06 (0.030)	6.15 (0.036)	10.34 (0.055)
*p*_0_	0.124	0.100	0.153	0.125 (0.0003)	0.099 (0.0005)	0.152 (0.0007)
σ (m)	18.5	16.3	21.0	18.7 (0.02)	16.5 (0.04)	21.4 (0.07)
*N* (lizards)	82.3	74.6	95.1	82.5 (0.11)	74.0 (0.10)	93.0 (0.26)

*N* is the abundance of lizards in the 9 ha search area. Lower and upper limits of 95% confidence intervals (based on 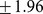 asymptotic standard errors) and of 95% credible intervals are given in the columns labeled 2.5% and 97.5%, respectively. Monte Carlo standard errors are given in parentheses.

### Simulation Study of Area-search Surveys

I repeated the simulation study of [7] and [26] to compare the operating characteristics of Bayes and empirical Bayes estimators of population abundance and average density based on the area-search models described in this paper. In the simulations a square (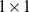 km) plot 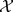 was searched for individuals on 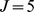 separate occasions. Individuals detected in each survey were marked (if unmarked) and released after noting their capture locations. Movements of individuals were assumed to follow a bivariate normal model with scale parameter 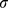 equal to 0.1 or 0.2 km. The region 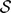 occupied by the population was assumed to be the square that buffers the survey plot 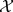 by 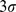 km on each side. The detection probability was assumed to be constant (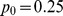) during each survey. The spatial distribution of individual activity centers was assumed to follow a homogeneous Poisson point process with limiting expected density 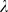 equal to 23.4, 46.9, or 78.1 individuals km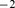.

To perform classical and Bayesian analyses of each simulated data set, I partitioned 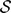 into a grid of 2500 square pixels for numerical integration. In the Bayesian analysis of each data set, I used the maximum-likelihood estimate of the model's parameters to initialize the Markov chain and I used 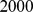 successive draws of the Gibbs sampler (Appendix S1) to estimate the model's parameters. Owing to the computational expense of these analyses, only 300 simulated data sets were analyzed for each combination of model parameters.

**Figure 7:**
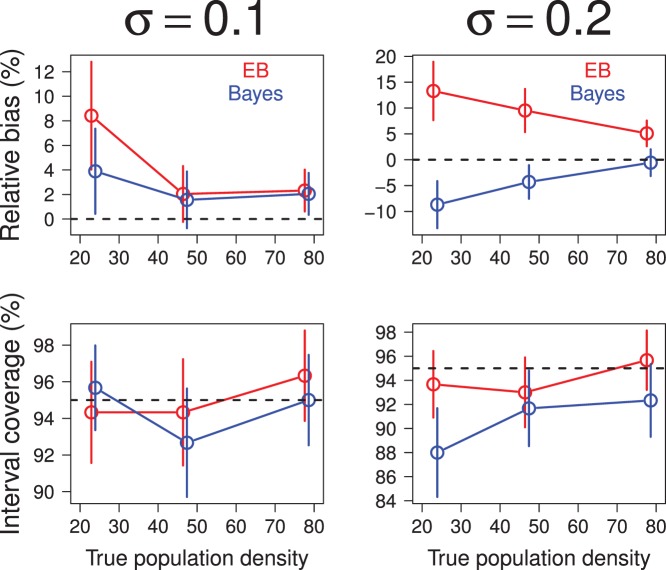
Comparison of emprical Bayes and Bayes estimates of population abundance computed from analyses of simulated recaptures in area-search surveys. Estimates of relative bias are plotted in upper row. Estimates of frequentist coverage of 95% credible or confidence intervals are plotted in lower row. Error bars indicate 95% confidence intervals.

**Figure 8:**
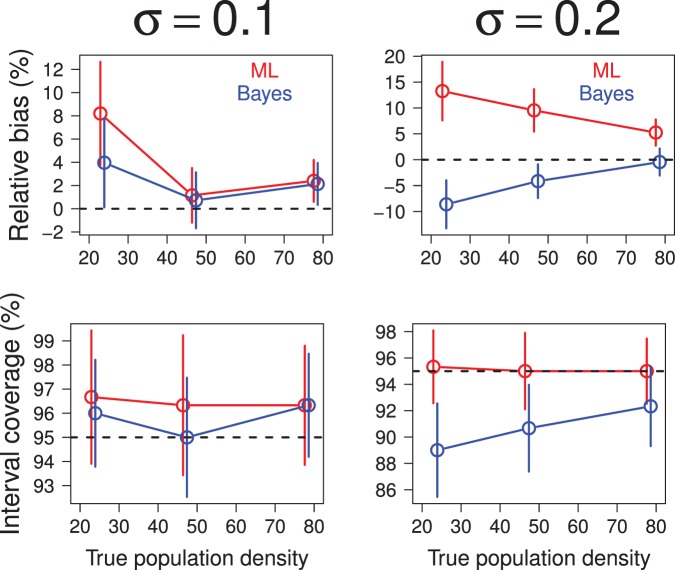
Comparison of maximum-likelihood and Bayes estimates of expected population density computed from analyses of simulated recaptures in area-search surveys. Estimates of relative bias are plotted in upper row. Estimates of frequentist coverage of 95% credible or confidence intervals are plotted in lower row. Error bars indicate 95% confidence intervals.

Classical and Bayes estimates of population abundance and average density were similar in most of the scenarios of the simulation study (Figures 7 and 8). The mean number of individuals observed ranged from about 20 individuals in the lowest density populations to about 67 or 74 individuals in the highest density populations (depending on the assumed value of 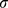). Estimates of relative bias of classical and Bayesian estimators were highest at the lowest population density and declined with increases in population density. The frequentist coverage of classical and Bayesian 95% confidence and credible intervals, respectively, was estimated to be at or near the nominal level in almost all simulation scenarios. One exception occurred at the lowest population density (23.4 individuals km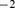) with 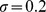, where the estimated coverage of Bayesian intervals was 0.88 (for abundance) and 0.89 (for density).

## Discussion

In formulating the SECR models I used a Poisson point process to specify the spatial distribution of individual activity centers. This modeling assumption allowed me to derive estimators of population abundance and density for use in classical or Bayesian analyses and to compare the operating characteristics of these estimators in analyses of real and simulated data sets.

The Bayes and empirical Bayes estimators of population abundance are closely related. Both estimators depend on the full conditional distribution of the number 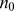 of activity centers of unobserved individuals (i.e., individuals not detected while sampling). This distribution is Poisson(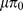) for *every* Poisson point-process model of SECR data. The only difference between models is the functional form of 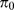, which depends on the samping protocol (e.g., compare (1) and (10)). In general, the empirical Bayes estimator of abundance equals the mean of the conditional distribution of 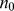 plus the observed number of individuals. However, since the mean of 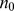 is a function of the model's parameters 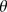, additional calculations are needed to ensure that the estimator of 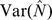 accounts for uncertainty involved in estimating 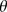 (see (5)). In contrast, the fully Bayesian estimator of population abundance is based on the marginal posterior distribution of the number of activity centers of unobserved individuals. This distribution is obtained by integrating the full conditional probabilities over the posterior distribution of 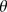 so that the Bayesian approach automatically accounts for uncertainty in estimating 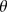.

An estimator of abundance proposed by [19] for the analysis of recaptures in trapping arrays is mathematically equivalent to the empirical Bayes estimator that I derived. In the notation of [19], 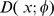 corresponds to 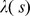 and 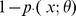 corresponds to 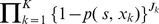. [19] also proposed three estimators of 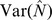; one of these (equation A2 of their appendix) appears to be equivalent to the estimator that I derived in Appendix S1. [19] presented this estimator without derivation, indicating only that it is based on an approximation of mean squared prediction error as described by [29]. I have shown that this estimator of 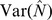 can be derived from an empirical Bayes view of the problem and is not an ad hoc approximation as suggested by [19]. In fact, the delta-method approximation of 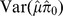 can be improved by parametric bootstrapping [25], though the additional calculations would rival those required for a fully Bayesian analysis using MCMC methods.

An empirical Bayes approach for estimating abundance is also feasible if a binomial point-process model is fitted to SECR data. In this case the conditional distribution of the number 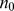 of activity centers of unobserved individuals is binomial with index parameter 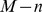 and success probability 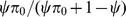 [17]. Therefore, an empirical Bayes estimator of 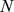 may be computed by adding 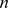 and the estimated conditional (binomial) mean of 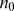. As with Poisson models, this approach can be used for every binomial model of SECR data. Only the functional form of 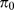 differs among models owing to differences in sampling protocols.

In the datasets I analyzed, classical and Bayesian methods provided similar – and often identical – inferences, which is not surprising given the sample sizes and the noninformative priors used in the analyses. For example, in the analysis of recaptures of tigers in trapping arrays and of lizards in area searches, classical and Bayesian estimates of the model parameters and their 95% confidence or credible intervals were quite similar and were consistent with previous analyses of these data [7], [17]. The same was true for Bayes and empirical Bayes estimates of abundance of these populations. In the analysis of recaptures from simulated populations, Bayesian estimates of abundance and average density exhibited levels of bias that were comparable to those of classical estimates (MLEs and empirical Bayes approximations). This result suggests that the differences in maximum-likelihood and Bayesian estimates reported by [7] were either statistically insignificant (due to high Monte Carlo error) or attributed to differences in modeling assumptions (binomial vs. Poisson).

The Bayesian SECR models that I described can be extended in a variety of useful and important ways. For example, the ecological process submodel can be extended to specify stochastic sources of spatial variation in density that induce clustering of individual activity centers (e.g., Cox process models). The Bayesian SECR models also can be revised to include spatial or non-spatial sources of variation in detectability of individuals or alternative functional forms for models of detectability [5]. The observation component of Bayesian SECR models also can be modified to account for different types of sampling methods (e.g., single- or multi-catch traps [5] or proximity detectors of acoustic signals [6]). I anticipate that these extensions of Bayesian SECR models will be developed in the near future.

## Supporting Information

Appendix S1
**Derivation of empirical Bayes estimator of Var(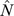), and MCMC algorithms used to fit Bayesian models of spatial capture-recapture data.**
(PDF)

## References

[1] Borchers D (2012) A non-technical overview of spatially explicit capture-recapture models. Journal of Ornithology 152: S435–S444.

[2] Efford M (2004) Density estimation in live-trapping studies. Oikos 106: 598–610.

[3] Borchers DL, Efford MG (2008) Spatially explicit maximum likelihood methods for capture- recapture studies. Biometrics 64: 377–385. 17970815 10.1111/j.1541-0420.2007.00927.x

[4] Dawson DK, Efford MG (2009) Bird population density estimated from acoustic signals. Journal of Applied Ecology 46: 1201–1209.

[5] Efford MG, Borchers DL, Byrom AE (2009) Density estimation by spatially explicit capture- recapture: likelihood-based methods. In: Thomson DL, Cooch EG, Conroy MJ, editors, Modeling demographic processes in marked populations, New York: Springer. 255–269.

[6] Efford MG, Dawson DK, Borchers DL (2009) Population density estimated from locations of individuals on a passive detector array. Ecology 90: 2676–2682. 19886477 10.1890/08-1735.1

[7] Efford MG (2011) Estimation of population density by spatially explicit capture-recapture analysis of data from area searches. Ecology 92: 2022–2207. 10.1890/11-0332.1 22352159

[8] Efford MG, Dawson DK, Robbins CS (2004) DENSITY: software for analyzing capture-recapture data from passive detector arrays. Animal Biodiversity and Conservation 27: 217–228.

[9] R Development Core Team (2012) R: A Language and Environment for Statistical Computing. R Foundation for Statistical Computing, Vienna, Austria. Available: http://www.R-project.org/. ISBN 3-900051-07-0.

[10] Royle JA, Young KV (2008) A hierarchical model for spatial capture-recapture data. Ecology 89: 2281–2289. 18724738 10.1890/07-0601.1

[11] Gardner B, Royle JA, Wegan MT (2009) Hierarchical models for estimating density from DNA mark-recapture studies. Ecology 90: 1106–1115. 19449704 10.1890/07-2112.1

[12] Royle JA, Karanth KU, Gopalaswamy AM, Kumar NS (2009) Bayesian inference in camera trapping studies for a class of spatial capture-recapture models. Ecology 90: 3233–3244. 19967878 10.1890/08-1481.1

[13] Royle JA, Nichols JD, Karanth KU, Gopalaswamy AM (2009) A hierarchical model for estimating density in camera-trap studies. Journal of Applied Ecology 46: 118–127.

[14] Gardner B, Reppucci J, Lucherini M, Royle JA (2010) Spatially explicit inference for open populations: estimating demographic parameters from camera-trap studies. Ecology 91: 3376–3383. 21141198 10.1890/09-0804.1

[15] Royle JA, Kéry M, Guélat J (2011) Spatial capture-recapture models for search-encounter data. Methods in Ecology and Evolution 2: 602–611.

[16] Sollmann R, Gardner B, Parsons AW, Stocking JJ, McClintock BT, et al (2013) A spatial mark- resight model augmented with telemetry data. Ecology 94: 553–559. 23687880 10.1890/12-1256.1

[17] Royle JA, Dorazio RM (2012) Parameter-expanded data augmentation for Bayesian analysis of capture-recapture models. Journal of Ornithology 152: S521–S537.

[18] Gopalaswamy AM, Royle JA, Hines JE, Singh P, Jathanna D, et al (2012) Program SPACECAP: software for estimating animal density using spatially explicit capture-recapture models. Methods in Ecology and Evolution 3: 1067–1072.

[19] Efford MG, Fewster RM (2013) Estimating population size by spatially explicit capture-recapture. Oikos 122: 918–928.

[20] Royle JA, Chandler RB, Sun CC, Fuller AK (2013) Integrating resource selection information with spatial capture-recapture. Methods in Ecology and Evolution 4: 520–530.

[21] Royle JA, Chandler RB, Sollmann R, Gardner B (2014) Spatial capture-recapture. Amsterdam: Academic Press.

[22] Cressie N, Wikle CK (2011) Statistics for spatio-temporal data. Hoboken, New Jersey: John Wiley & Sons.

[23] Gelfand AE, Smith AFM (1990) Sampling-based approaches to calculating marginal densities. Journal of the American Statistical Association 85: 398–409.

[24] Royle JA, Dorazio RM (2008) Hierarchical modeling and inference in ecology. Amsterdam: Academic Press.

[25] Laird NM, Louis TA (1987) Empirical Bayes confidence intervals based on bootstrap samples (with discussion). Journal of the American Statistical Association 82: 739–757.

[26] Marques TA, Thomas L, Royle JA (2011) A hierarchical model for spatial capture-recapture data: comment. Ecology 92: 526–528. 21618931 10.1890/10-1440.1

[27] Flegal JM, Jones GL (2010) Batch means and spectral variance estimators in Markov chain Monte Carlo. Annals of Statistics 38: 1034–1070.

[28] Flegal JM, Jones GL (2011) Implementing MCMC: estimating with confidence. In: Brooks S, Gelman A, Jones GL, Meng XL, editors, Handbook of Markov chain Monte Carlo, Boca Raton, Florida: Chapman & Hall/CRC. 175–197.

[29] Johnson DS, Laake JL, Ver Hoef JM (2010) A model-based approach for making ecological inference from distance sampling data. Biometrics 66: 310–318. 19459840 10.1111/j.1541-0420.2009.01265.x

