# Supplementary material for: Bayes and Empirical Bayes Estimators of Abundance and Density from Spatial Capture-Recapture Data
Source: PLoS One. 2013 Dec 27;8(12):e84017. doi: 10.1371/journal.pone.0084017 (PMC3873963; doi:10.1371/journal.pone.0084017)
Supplement: Appendix S1 — Derivation of empirical Bayes estimator of Var(), and MCMC algorithms used to fit Bayesian models of spatial capture-recapture data. (PDF) [file pone.0084017.s001.pdf]

Appendix S1

Derivation of empirical Bayes estimator of Var($\hat{N}$)

In this appendix I derive an empirical Bayes estimator of Var($\hat{N}$) that accounts for the expected variance of n_0 based on its conditional distribution (Poisson($\mu\pi_0$)) and for the uncertainty involved in estimating $\boldsymbol{\theta}$.

The derivation is motivated by a fully Bayesian approach wherein all inferences about n_0 (and thus about $N = n + n_0$) are based on its marginal posterior distribution, which has mass function

$$[n_0|\boldsymbol{Y}_{\text{obs}}, n] = \int [n_0|\boldsymbol{Y}_{\text{obs}}, n, \boldsymbol{\theta}]\ [\boldsymbol{\theta} \mid \boldsymbol{Y}_{\text{obs}}, n]\, d\boldsymbol{\theta}$$

(as shown in (6)). In a fully Bayesian approach the posterior variance of n_0 (and of N) is the sum of two components that account for the posterior uncertainty of $\boldsymbol{\theta}$:

$$\text{Var}(n_0|\boldsymbol{Y}_{\text{obs}}, n) = \text{E}_{\boldsymbol{\theta}|\boldsymbol{Y}_{\text{obs}},n}\{\text{Var}(n_0|\boldsymbol{Y}_{\text{obs}}, n, \boldsymbol{\theta})\} + \text{Var}_{\boldsymbol{\theta}|\boldsymbol{Y}_{\text{obs}},n}\{\text{E}(n_0|\boldsymbol{Y}_{\text{obs}}, n, \boldsymbol{\theta})\}$$

In contrast, when using an empirical Bayes approach, $\boldsymbol{\theta}$ is assumed to be constant and inferences are based on the distribution of $\hat{\boldsymbol{\theta}}$ under a sequence of hypothetical repeated samples.

Following this classical view, [1] proposed an empirical Bayes approach for approximating $\text{Var}(n_0|\boldsymbol{Y}_{\text{obs}}, n)$ based on the distribution of $\boldsymbol{\theta}$ estimates that would arise if an arbitrarily large number of independent data sets were generated (from the model's assumptions and the estimate $\hat{\boldsymbol{\theta}}$) and if each of these data sets was used to compute an estimate of $\boldsymbol{\theta}$, say $\tilde{\boldsymbol{\theta}}$. Let $\tilde{\boldsymbol{\theta}}|\hat{\boldsymbol{\theta}}$ denote the sampling distribution of these estimates. The proposal of [1] is to approximate the posterior uncertainty in $\boldsymbol{\theta}$ by substituting $\tilde{\boldsymbol{\theta}}|\hat{\boldsymbol{\theta}}$ for the marginal posterior $\boldsymbol{\theta}|\boldsymbol{Y}_{\text{obs}}, n$. In the context of our estimation problem, this approach leads to the following approximation:

$$\text{Var}(n_0|\boldsymbol{Y}_{\text{obs}}, n) \doteq \text{E}_{\tilde{\boldsymbol{\theta}}|\hat{\boldsymbol{\theta}}}\{\text{Var}(n_0|\boldsymbol{Y}_{\text{obs}}, n, \tilde{\boldsymbol{\theta}})\} + \text{Var}_{\tilde{\boldsymbol{\theta}}|\hat{\boldsymbol{\theta}}}\{\text{E}(n_0|\boldsymbol{Y}_{\text{obs}}, n, \tilde{\boldsymbol{\theta}})\}$$

Parametric bootstrapping, the approach advocated by [1], provides one method for calculating this approximation of $\text{Var}(n_0|\boldsymbol{Y}_{\text{obs}}, n)$; however, in our problem this approach is computationally intensive and rivals the number of calculations required for a fully Bayesian analysis using MCMC methods. Therefore, in the spirit of empirical Bayes I propose the following plug-in estimator:

$$\begin{aligned}\widehat{\text{Var}}(n_0|\boldsymbol{Y}_{\text{obs}}, n) &= \text{Var}(n_0|\boldsymbol{Y}_{\text{obs}}, n, \hat{\boldsymbol{\theta}}) + \text{Var}\{\text{E}(n_0|\boldsymbol{Y}_{\text{obs}}, n, \hat{\boldsymbol{\theta}})\} \\ &= \hat{\mu}\hat{\pi}_0 + \text{Var}(\hat{\mu}\hat{\pi}_0) \\ &= \hat{\mu}\hat{\pi}_0 + \boldsymbol{\nabla}(\hat{\mu}\hat{\pi}_0)'\,\widehat{\text{Var}}(\hat{\boldsymbol{\theta}})\,\boldsymbol{\nabla}(\hat{\mu}\hat{\pi}_0)\end{aligned}$$

which is based on a delta-method approximation of $\text{Var}(\hat{\mu}\hat{\pi}_0)$ in which $\widehat{\text{Var}}(\hat{\boldsymbol{\theta}})$ denotes the estimated asymptotic variance-covariance matrix of $\hat{\boldsymbol{\theta}}$ and $\boldsymbol{\nabla}(\hat{\mu}\hat{\pi}_0)$ denotes the gradient of $\mu\pi_0$ with respect to $\boldsymbol{\theta}$ evaluated at $\hat{\boldsymbol{\theta}}$.

In summary, I denote the empirical Bayes estimator of N by $\hat{N} = n + \hat{\mu}\hat{\pi}_0$ and the empirical Bayes estimator of $\text{Var}(\hat{N})$ by $\widehat{\text{Var}}(\hat{N}) = \hat{\mu}\hat{\pi}_0 + \boldsymbol{\nabla}(\hat{\mu}\hat{\pi}_0)'\,\widehat{\text{Var}}(\hat{\boldsymbol{\theta}})\,\boldsymbol{\nabla}(\hat{\mu}\hat{\pi}_0)$.

MCMC algorithms used to fit Bayesian models of spatial capture-recapture data

Model of Recaptures at Trapping Array Locations

To compute summaries of the posterior distribution, I used a Gibbs sampling algorithm based on the following posterior density function:

$$[\boldsymbol{\theta}, n_0 \mid \boldsymbol{Y}_{\text{obs}}, n] = C \; [\boldsymbol{Y}_{\text{obs}}, n, n_0|\boldsymbol{\theta}] \; [\boldsymbol{\theta}]$$

where $\boldsymbol{\theta} = (\boldsymbol{\beta}, p_0, \sigma)'$, C denotes the normalizing constant of the posterior, and $[\boldsymbol{Y}_{\text{obs}}, n, n_0|\boldsymbol{\theta}]$ is the complete-data likelihood function. (In this appendix, I use C generically to denote the normalizing constant of a distribution.) I assume mutually independent priors for the parameters in $\boldsymbol{\theta}$ (i.e., $[\boldsymbol{\theta}] = [\boldsymbol{\beta}][p_0][\sigma]$).

The Gibbs sampler is based on the following full-conditional distributions:

1. The full conditional for n_0 follows directly from (3); therefore, $n_0|\cdot \sim \text{Poisson}(\mu\pi_0)$ where π_0 is defined in (1).

2. Assuming a normal (Gaussian) prior distribution for each element of $\boldsymbol{\beta}$ leads to full conditionals that do not have a simple form. In principle, these distributions may be sampled using Metropolis or Metropolis-Hastings algorithms with target density

$$[\boldsymbol{\beta}|\cdot] = C \; [\boldsymbol{Y}_{\text{obs}}, n, n_0|\boldsymbol{\beta}, p_0, \sigma] \; [\boldsymbol{\beta}].$$

I used this approach for each element of $\boldsymbol{\beta}$ except β_0. Specifically, I assumed a $N(0, \tau^2)$ prior for each element where the prior variance τ^2 was assigned to be arbitrarily large, thereby specifying high prior uncertainty. To sample the full conditional of each element, I used a random-walk Metropolis sampler with the full conditional density of β_j ($j > 0$) as target and with a normal proposal distribution. The variance parameter of this proposal was tuned adaptively by incrementing or decrementing the proposal variance depending on whether or not the acceptance rate in each batch of 50 iterations of the Gibbs sampler exceeded a target rate of 0.44 [2]. I reduced the absolute value of these adjustments in proportion to the inverse square root of the number of batches to ensure that the diminishing-adaptation condition required for convergence (in distribution) of the Markov chain was satisfied [3].

For the intercept parameter β_0, I assumed a $\text{Gamma}(\alpha, \alpha/\lambda_0)$ prior distribution for $\lambda = \exp(\beta_0)$ that has mean λ_0 and variance λ_0^2/α. This prior was selected because λ corresponds to the (limiting) expected density of individuals at the average value of the covariates. In the absence of covariates the gamma prior would imply a negative-binomial prior for N with mean $\lambda_0 A(\mathcal{S})$ and variance $\lambda_0 A(\mathcal{S}) + \{\lambda_0 A(\mathcal{S})\}^2/\alpha$. More importantly, the gamma prior would imply a full conditional of simple form:

$$\lambda|\cdot \sim \text{Gamma}(\alpha + n_0 + n, \; \alpha/\lambda_0 + A(\mathcal{S}))$$

Therefore, if little is known about the magnitude of λ in advance of collecting the data, we can assign high prior variance by making α arbitrarily small. For a homogenous Poisson point-process model, this assumption leads to estimates of λ that are dominated by the data because $\text{E}(\lambda|\cdot) \to (n_0 + n)/A(\mathcal{S})$ as $\alpha \to 0$. Conceptually, these benefits are inherited by the full conditional of β_0; however, owing to the reparameterization (from β_0 to λ), the conditional density does not have a familiar form:

$$[\beta_0|\cdot] = C \; \exp\left[\alpha\big[\beta_0 - \log(\lambda_0) - \exp\{\beta_0 - \log(\lambda_0)\}\big]\right]$$

Thus, to sample the full conditional of β_0, I used a random-walk Metropolis sampler with the full conditional density of β_0 as target and with a normal proposal distribution. The variance parameter of the proposal was tuned adaptively as described earlier.

3. I assumed a uniform prior distribution for p_0, but I estimated this parameter on the logit scale: $\eta_0 = \log\{p_0/(1-p_0)\}$. Therefore, the prior density of this parameter is $[\eta_0] = \exp(\eta_0)/\{1+\exp(\eta_0)\}^2$, and the density of its full conditional distribution is

$$[\eta_0|\cdot] = C\ [\eta_0]\, \pi_0^{n_0} \prod_{i=1}^{n} \int_{\mathcal{S}} \lambda(\boldsymbol{s}_i) \prod_{k=1}^{K} \mathrm{Bin}(y_{ik}|J_k, p(\boldsymbol{s}_i, \boldsymbol{x}_k))\, d\boldsymbol{s}_i$$

   To sample the full conditional of η_0, I used a random-walk Metropolis sampler with the full conditional density of η_0 as target and with a normal proposal distribution. The variance parameter of the proposal was tuned adaptively as described earlier.

4. For the variance parameter σ, I assumed a half-Cauchy prior [4] with unit scale parameter. [4] showed that this prior avoids problems that can occur when alternative "noninformative" priors are used (including the nearly improper, Inverse-Gamma(ϵ, ϵ) family). I estimated σ on the log scale: $\phi = \log(\sigma)$. Therefore, the prior density of this parameter is $[\phi] = 2\exp(\phi)/[\pi\{1 + \exp(2\phi)\}]$, and the density of its full conditional distribution is

$$[\phi|\cdot] = C\ [\phi]\, \pi_0^{n_0} \prod_{i=1}^{n} \int_{\mathcal{S}} \lambda(\boldsymbol{s}_i) \prod_{k=1}^{K} \mathrm{Bin}(y_{ik}|J_k, p(\boldsymbol{s}_i, \boldsymbol{x}_k))\, d\boldsymbol{s}_i$$

   To sample the full conditional of ϕ, I used a random-walk Metropolis sampler with the full conditional density of ϕ as target and with a normal proposal distribution. The variance parameter of the proposal was tuned adaptively as described earlier.

Model of Recapture Locations Observed in Area Searches

To compute summaries of the posterior distribution, I used a Gibbs sampling algorithm based on the following posterior density function:

$$[\boldsymbol{\theta}, n_0 \mid \boldsymbol{Y}_{\mathrm{obs}}, \boldsymbol{X}_{\mathrm{obs}}, n] = C\ [\boldsymbol{Y}_{\mathrm{obs}}, \boldsymbol{X}_{\mathrm{obs}}, n, n_0|\boldsymbol{\theta}]\ [\boldsymbol{\theta}]$$

where $\boldsymbol{\theta} = (\boldsymbol{\beta}, p_0, \sigma)'$ and $[\boldsymbol{Y}_{\mathrm{obs}}, \boldsymbol{X}_{\mathrm{obs}}, n, n_0|\boldsymbol{\theta}]$ is the complete-data likelihood function. I assume mutually independent priors for the parameters in $\boldsymbol{\theta}$ (i.e., $[\boldsymbol{\theta}] = [\boldsymbol{\beta}][p_0][\sigma]$).

The Gibbs sampler is similar to that developed for the previous model, in part, because the same priors and reparameterizations of $\boldsymbol{\theta}$ are used. The Gibbs sampler is based on the following full-conditional distributions:

1. The full conditional for n_0 follows directly from (3); therefore, $n_0|\cdot \sim$ Poisson$(\mu\pi_0)$ where π_0 is defined in (10).

2. As described earlier, for each element of $\boldsymbol{\beta}$ except β_0 I used a random-walk Metropolis algorithm with target densities based on

$$[\boldsymbol{\beta}|\cdot] = C\ [\boldsymbol{Y}_{\mathrm{obs}}, \boldsymbol{X}_{\mathrm{obs}}, n, n_0|\boldsymbol{\beta}, p_0, \sigma]\ [\boldsymbol{\beta}]$$

   To sample the full conditional of β_0, I used a random-walk Metropolis sampler with the full conditional density of β_0

$$[\beta_0|\cdot] = C\ \exp\left[\alpha\big[\beta_0 - \log(\lambda_0) - \exp\{\beta_0 - \log(\lambda_0)\}\big]\right]$$

as target and with a normal proposal distribution. The variance parameter of the proposal was tuned adaptively as described earlier.

3. The density of the full conditional distribution of η_0 (= logit(p_0)) is

$$[\eta_0|\cdot] = C\ [\eta_0]\,\pi_0^{n_0}\prod_{i=1}^{n}\int_{\mathcal{S}}\lambda(\boldsymbol{s}_i)\left\{\prod_{j:y_{ij}=1}\mathrm{N}(\boldsymbol{x}_{ij}|\boldsymbol{s}_i,\sigma^2\boldsymbol{I})\ p_0\right\}\left\{1-\int_{\mathcal{X}}\mathrm{N}(\boldsymbol{x}|\boldsymbol{s}_i,\sigma^2\boldsymbol{I})\,p_0\,d\boldsymbol{x}\right\}^{J-y_{i\cdot}}d\boldsymbol{s}_i$$

To sample the full conditional of η_0, I used a random-walk Metropolis sampler with the full conditional density of η_0 as target and with an adaptively tuned normal proposal distribution, as described for the previous model.

4. The density of the full conditional distribution of ϕ (= log(σ)) is

$$[\phi|\cdot] = C\ [\phi]\,\pi_0^{n_0}\prod_{i=1}^{n}\int_{\mathcal{S}}\lambda(\boldsymbol{s}_i)\left\{\prod_{j:y_{ij}=1}\mathrm{N}(\boldsymbol{x}_{ij}|\boldsymbol{s}_i,\sigma^2\boldsymbol{I})\ p_0\right\}\left\{1-\int_{\mathcal{X}}\mathrm{N}(\boldsymbol{x}|\boldsymbol{s}_i,\sigma^2\boldsymbol{I})\,p_0\,d\boldsymbol{x}\right\}^{J-y_{i\cdot}}d\boldsymbol{s}_i$$

To sample the full conditional of ϕ, I used a random-walk Metropolis sampler with the full conditional density of ϕ as target and with an adaptively tuned normal proposal distribution, as described for the previous model.

References

1. Laird NM, Louis TA (1987) Empirical Bayes confidence intervals based on bootstrap samples (with discussion). Journal of the American Statistical Association 82: 739–757.

2. Rosenthal JS (2011) Optimal proposal distributions and adaptive MCMC. In: Brooks S, Gelman A, Jones GL, Meng XL, editors, Handbook of Markov chain Monte Carlo, Boca Raton, Florida: Chapman & Hall / CRC. pp. 93–111.

3. Roberts GO, Rosenthal JS (2007) Coupling and ergodicity of adaptive Markov chain Monte Carlo algorithms. Journal of Applied Probability 44: 458–475.

4. Gelman A (2006) Prior distributions for variance parameters in hierarchical models (Comment on article by Browne and Draper). Bayesian Analysis 1: 515–534.
